# Baby Boomers’ Adoption of Consumer Health Technologies: Survey on Readiness and Barriers

**DOI:** 10.2196/jmir.3049

**Published:** 2014-09-08

**Authors:** Cynthia LeRouge, Craig Van Slyke, Deborah Seale, Kevin Wright

**Affiliations:** ^1^College for Public Health and Social JusticeDepartment of Health Management & PolicySaint Louis UniversitySaint Louis, MOUnited States; ^2^W.A. Franke College of BusinessNorthern Arizona UniversityFlagstaff, AZUnited States; ^3^Health Informatics and Information ManagementSaint Louis UniversitySaint Louis, MOUnited States; ^4^College of Humanities and Social SciencesCommunications DepartmentGeorge Mason UniversityFairfax, VAUnited States

**Keywords:** baby boomer, readiness, adoption, consumer health technology, man-machine systems, aging, health, human factors, design, user interfaces, personal computing

## Abstract

**Background:**

As they age, baby boomers (born 1946-1964) will have increasing medical needs and are likely to place large demand on health care resources. Consumer health technologies may help stem rising health care needs and costs by improving provider-to-patient communication, health monitoring, and information access and enabling self-care. Research has not explored the degree to which baby boomers are ready for, or are currently embracing, specific consumer health technologies This study explores how baby boomers’ readiness to use various technologies for health purposes compares to other segments of the adult population.

**Objective:**

The goals of the study are to (1) examine what technologies baby boomers are ready to use for health purposes, (2) investigate barriers to baby boomers’ use of technology for health purposes, and (3) understand whether readiness for and barriers to baby boomers’ use of consumer health technologies differ from those of other younger and older consumers.

**Methods:**

Data were collected via a survey offered to a random sample of 3000 subscribers to a large pharmacy benefit management company. Respondents had the option to complete the survey online or by completing a paper-based version of the survey.

**Results:**

Data from 469 respondents (response rate 15.63%) were analyzed, including 258 baby boomers (aged 46-64 years), 72 younger (aged 18-45 years), and 139 older (age >64 years) participants. Baby boomers were found to be similar to the younger age group, but significantly more likely than the older age group to be ready to use 5 technologies for health purposes (health information websites, email, automated call centers, medical video conferencing, and texting). Baby boomers were less ready than the younger age group to adopt podcasts, kiosks, smartphones, blogs, and wikis for health care purposes. However, baby boomers were more likely than older adults to use smartphones and podcasts for health care purposes. Specific adoption barriers vary according to the technology.

**Conclusions:**

Baby boomers have commonalities with and distinctions from both younger and older adults in their readiness to adopt specific consumer health technologies and the barriers they experience to adoption. Baby boomers’ nuances regarding readiness to adopt and the barriers associated with the various forms of consumer health technology should be taken into account by those interested in promoting consumer health technologies use among baby boomers when developing applications, choosing technologies, preparing users for use, and in promotional tactics.

## Introduction

### Background

 The United States health care system faces a coming “gray tsunami” as baby boomers (those born between 1946 and 1964) begin requiring more health care resources [1,2]. The United States is one of many countries around the world that are experiencing new demands on their health care systems due to a rise in the number of older adults and health conditions associated with aging [3-5]. According to a report by the American Hospital Association and First Consulting Group [6], baby boomers make up the largest segment of the population in the United States (approximately 78 million Americans based on the 2000 US Census) [7,8]. In 2011, the first members of the baby boom generation reached age 65. As baby boomers reach retirement age, many will have increasing medical needs and thus demand more health care resources than other segments of the population, particularly given the size of this age cohort and that their life expectancies are longer than many past generations [6]. Age is often associated with growing health problems and chronic disease [9]. Approximately 60% of baby boomers have already been diagnosed with at least 1 chronic medical condition. Arthritis, diabetes, heart disease, obesity, osteoporosis, and hypertension are common chronic conditions among baby boomers. These conditions require regular health care checkups, prescription medications, and significant dietary changes [6]. Given the size of the baby boomer cohort, responding to age-related health concerns will likely be a significant challenge for health care systems over the next decade and beyond [10].

The health care industry must be prepared to accommodate this growing segment of health care consumers. Similar to previous generations, many baby boomers will require extensive health services, training and reinforcement for medical self-management, as well as continued connection to clinicians and contact with their peers and caregivers as they grow older [11]. It is questionable whether the traditional US health care system can handle the level of health care demands imposed by the gray tsunami given the immense size of the baby boomer cohort compared to previous older generations [1]. If empowerment leads to an increase in self-care, patient empowerment efforts aimed at prevention and self-management of chronic disease could play a key role in relaxing some of the demands on the health care system [12]. Given current changes in the health care marketplace and the need to find more cost-efficient ways to manage health conditions, baby boomers will likely be the generation that leads the movement toward patient self-management of chronic disease [13].

Emerging health information and communication technologies bring the promise of transformations in the delivery of care, empowering patients to make more informed health care decisions, connecting patients directly to providers and other caregivers and personalizing services in response to patients’ unique needs and preferences [13]. Information and communication technologies used by consumers for health purposes are increasingly allowing individuals to conveniently learn about, manage, and monitor their health via electronic devices. The use of consumer health technologies may help stem rising health care costs by improving provider-to-patient communication, health condition monitoring, and health information access by enabling self-care [14,15]. Baby boomers may differ from older cohorts in terms of their exposure to new technologies (e.g, being exposed to new technologies in the workplace). Because baby boomers will be the largest older adult cohort in history, it is important to assess the barriers to consumer health technologies within this population; consumer health technologies will likely continue to be a means of controlling health care costs in the face of growing demands on the health care system (due to its increased use by baby boomers as they begin to face health issues associated with aging).

But, are baby boomers currently embracing or even ready to use modern technologies to manage their health? Although baby boomers may have more technology access and experience than prior generations, it is not clear if this leads to greater readiness to adopt consumer health technologies. For example, researchers have found that although barriers exist for some technologies, older consumers are ready and able to adopt other technologies for health-related tasks [16-19]. Designing and developing consumer health technologies that effectively meet the unique requirements of baby boomers requires understanding which types of consumer health technologies baby boomer consumers are ready to adopt and what barriers exist for the specific technologies where readiness is low. Previous research has not adequately addressed these issues from the perspective of baby boomers.

The purpose of this study is to address the following research goals. First, we examine what consumer health technologies baby boomers are ready to use. Second, we investigate barriers to baby boomers’ use of consumer health technologies. Finally, we seek to understand whether readiness for and barriers to baby boomers’ use of consumer health technologies differ from those for other consumers (younger and older). Gaining a better understanding of these issues will help proponents of consumer health technology better understand how to build and promote systems that bring about benefits to baby boomer consumers. This is especially noteworthy because baby boomers may become a driving force behind the development of consumer health technologies given the demands on the health care system they are predicted to create.

### Theoretical Framework

Consumer health technologies attempt to engage health consumers to interact with technology to promote healthy behaviors and informed decision making. The Agency for Healthcare Research and Quality notes consumer health information technology (IT) applications are a key topic and indicate that:

These [consumer health technology] applications have various purposes including assisting with self-management through reminders and educational prompts, delivering real-time data on a patient’s health condition to both patients and providers, facilitating Web-based support groups, and compiling and storing personal health information in an easily accessible format...Moreover, consumer health IT applications that allow gathering and integrating data from various health care sources can serve as a comprehensive resource for patients and their providers. In addition to convenience, consumer health IT applications also can be important in emergency situations to provide critical health information to medical staff. [20]

**Figure 1:**
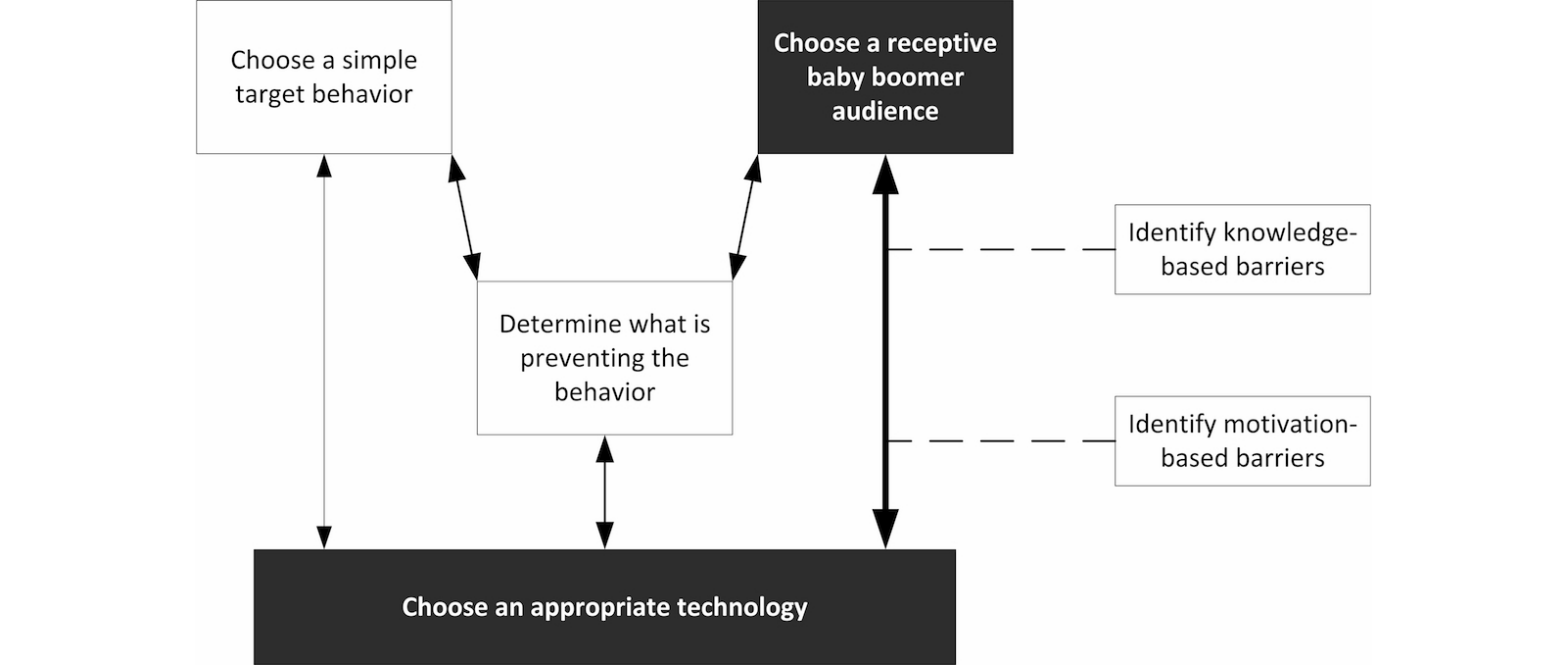
Phase 1 of steps in the persuasive design process (adapted from Fogg [24]). Areas of focus for the current study are highlighted in black and gray.

Most consumer health technologies are designed to change attitudes or behaviors and provide information. The effectiveness of consumer health technologies requires choosing a receptive audience and an appropriate technology [21-23]. Persuasive technology design can facilitate coaxing the user toward healthy action (motivating factor) and underscore the need to choose a receptive audience and befitting technology [22-24]. A model of the persuasive design process (drawn from demonstrated success in industries including health care) begins with defining the persuasion goal to match a receptive target audience with an appropriate technology (Figure 1 depicts this first stage of the persuasive design process; areas of focus for the current study are shaded) [24]. This type of alignment coincides with modern human-computer interaction design philosophy in which the needs, desires, and limitations of users are investigated and analyzed [25]. If various forms of consumer health technology are to be successful, we must have a fundamental understanding of which technology tools align with baby boomers’ needs, desires, and limitations. Indeed, recent research focused on physical and psychological attributes as 1 of 4 types of patient barriers to eHealth opportunity (the other 3 types include the provision of eHealth opportunity, the support others that may have to use eHealth, and economic barriers) [26].

Unfortunately, the literature that specifically focuses on adoption of consumer health technologies and its use by baby boomers is limited. Some noted exceptions relate to home monitoring devices. For example, work by Mihailidis and colleagues [27] found a general willingness of current baby boomers to accept various forms of home monitoring technology (eg, personal emergency response systems, fall detection systems). However, it is important to note that the Mihailidis et al study was largely exploratory and it was limited by a small sample size [27]. In addition, a study of Australian baby boomers found baby boomers were generally open to use assistive technologies for a temporary period after hospital discharge [28]. In both studies, the home monitoring and assistive technologies studied were quite passive as compared to the interactive, multipurpose technologies frequently used in consumer health informatics, such as the Internet and smartphones. We know of no study that specifically explores various common interactive technology tools for consumer health informatics use by baby boomers. It is from this need that we provide our first research question:

Research question 1: What technologies are baby boomers ready to use to promote healthy behaviors and informed decision making?

As part of that foundational understanding of which consumer health technology tools align with baby boomers’ needs, desires, and limitations, we must recognize that baby boomers are different from younger and older consumers in many ways, 2 of which are particularly important to note. First, baby boomers are not the “digital native” youths, who have known these technologies their entire lives, nor are they like their elders, most of whom had little exposure to interactive information technologies in their work lives. Many baby boomers experienced the transition to more of an information technology workforce. There might be some social bias that those who are not digital natives may not be as open to consumer health technologies. However, recent studies indicate that this is not the case. A recent study found baby boomers and older adults were generally open to home monitoring devices and did not have strong preferences with respect to the types and locations of the technology [27]. But, do these similarities extend to more interactive technologies? Unlike their predecessors, many baby boomers are comfortable with interactive technology [29,30]. But, unlike their successors, many baby boomers do not naturally turn to technology as their first choice when communicating, seeking information, or looking for task support for health needs [11]. For example, according to the Pew Research Center’s 2013 update on smartphone ownership [31], only 39% of those surveyed aged 55-64 years owned a smartphone. This percentage was even lower (18%) for those 65 years or older. Younger consumers reported much higher ownership percentages [31]. For example, 81% of those aged 25-34 years reported owning a smartphone [31].

The second important difference relates to baby boomers’ increased expectations concerning health care services [6]. Because aging baby boomers have higher levels of education, more disposable income in terms of being in their peak earning years compared to younger and older age cohorts (although overall income and savings may be affected by numerous variables, including life circumstances and the recent economic downturn), and are more active than previous generations, baby boomers are naturally more focused on health care services that ensure their long-term mobility and independence [32]. The higher expectations of baby boomers are reflected in increased demands for innovative and personalized health care services that eliminate barriers to treatment and provide timely and accurate health-related information and services. In addition, many baby boomers are now caring for elderly parents, while trying to maintain an active lifestyle, which further increases their health information and service needs [33]. To further explore how baby boomers’ readiness for various consumer health technology tools compares to other segments of the adult population, we introduce the following research question:

Research question 2: How do the technology tools that baby boomers report they are ready to use for health purposes differ from the technology tools younger adults and older adults report that they are ready to use for health purposes?

Prior research indicates that baby boomers may face a number of barriers in adopting consumer health technologies [34,35]. Innovation diffusion theory [36] provides a useful theoretical framework for investigating the “internal” barriers particular to the personal decision process of adoptions (in contrast to external factors such as provision of eHealth opportunity and economic barriers). Rogers [36] posits that potential innovation adopters go through a 5-stage process when deciding whether to adopt and use an innovation: knowledge, persuasion, decision, implementation, and confirmation. The first 2 stages, knowledge and persuasion, are of primary interest to this study and require additional explication (note that the discussion of the stages is based on Rogers [36] unless otherwise indicated).

In the knowledge stage, the potential adopter becomes aware that an innovation exists. This awareness is followed by the adopter forming an understanding of how the innovation functions. Two important sets of information related to the adopter are important to this stage. First, prior conditions, such as prior experience with similar innovations, problems faced by the adopter, and social system norms, impact the knowledge stage. For example, problems faced by the adopter that may be met by the innovation’s use are likely to influence how the adopter frames knowledge about the innovation. Prior experience with similar innovations may likewise influence knowledge of the innovation. For example, a consumer who has experience with a smartphone will build knowledge of a tablet computer differently than a consumer without such prior experience. Characteristics of the adopter are also important in the knowledge stage. This is particularly important for our research given that age is an essential individual characteristic related to innovation adoption [37].

In the persuasion stage, the potential adopter forms attitudes related to the innovation and its use. (It is important to note that Rogers defines *persuasion* as the formation of attitudes rather than a change agent’s activities to influence those attitudes.) Perceptions regarding the innovation’s attribute use are the building blocks of the adopter’s innovation-related attitudes. Generally, the adopter is concerned with advantages and disadvantages of the innovation, given the adopter’s particular situation.

During the decision stage, the adopter makes the choice to adopt or reject an innovation. Note that adoption is the decision to make use of the innovation, not the actual use of the innovation. Use occurs in the implementation stage. Use represents an explicit behavioral change that puts the innovation into practice. Post implementation information seeking intended to reinforce the already-made innovation decision follows the implementation stage. In Rogers’ model, this is known as the confirmation stage. The confirmation stage may result in continuation or reversal of the prior innovation decision.

In this study, we are interested in 2 types of barriers to consumers’ use of technology: knowledge-based barriers and motivation-based barriers. We acknowledge that we are making an implicit assumption that the consumer has material access to the technology. In other words, the consumer has the means to physically possess the technology and necessary network access [38]. Our research model (presented in Figure 1) recognizes these 2 categories of barriers to the adoption and use of consumer health technologies. Knowledge-based barriers concern a lack of knowledge of the technology’s existence, purpose, and operation. Motivation-based barriers relate to beliefs about the benefits of using the technology relative to the drawbacks of using the technology. Knowledge and motivation barriers align with the first 2 stages of Rogers’ [36] innovation-decision process: knowledge and persuasion. During the knowledge stage, consumers become aware of the innovation and begin to understand its uses. In the persuasion stage, consumers form beliefs about the technology and its uses. Individual characteristics, such as age, impact both knowledge of and beliefs about a technology [36]. Therefore, we believe it will be instructive to examine baby boomers’ awareness and perceptions of various consumer health technologies.

We first address barriers related to knowledge. The most fundamental of these is a lack of awareness of the technology and its uses [34]. Awareness may partially explain differences between different age cohorts when it comes to the adoption of the new communication technologies. For example, baby boomers are much more likely to own smartphones, desktop computers, and laptop computers than older cohorts, but they are somewhat less likely to own newer technologies (such as iPads) or use certain applications, such as using a smartphone to send/receive emails or access the Internet, than younger cohorts [30,39,40]. Knowledge barriers beyond awareness also exist. Consumers may be aware of the technology, but lack knowledge of its purpose or its operation. For example, consumers may be aware that kiosks exist, but may not know what they can be used for or how to use them.

The second category of barriers relates to motivations to adopt the technology. These barriers concern beliefs about the costs and benefits of using a technology for a specific purpose. For example, making consumer health technologies available for baby boomers with different cognitive, perceptual, and physical abilities is challenging [35,41] because these differences change the cost-benefit calculus. Moreover, baby boomer perceptions of the usefulness and usability of various consumer health technologies, the efficiency of care delivery, cost, and improvement of quality of life stemming from the use of these technologies may be barriers that inhibit adoption and use [28,35].

In the persuasion stage of the innovation adoption decision, the consumer begins to form beliefs about the technology as it relates to health care information acquisition and use. This is the beginning of an adoption cost-benefit evaluation by the consumer that determines the outcome of the adoption decision [42]. Because our primary interest here is in barriers, we focus on the adoption cost side of the equation. Several adoption costs are of particular interest to consumer health technologies. First, the relative difficulty of using the consumer health technology may serve as a barrier to its use. The relationship between perceived complexity and the use of an innovation is well established [43-46]. In some cases, training may reduce the effort required to use the technology. However, in other cases, the user may already know how to use the technology (ie, is trained), but may feel that the effort required to put the technology into use is too high. These barriers align with the van Dijk and Hacker’s [38] concept of skills access, which indicates that a lack of digital skills causes a combination of inadequate training and high complexity.

Beliefs regarding the suitability of a technology for health information tasks are also important. When a consumer perceives a technology unsuitable for use (not compatible with particular uses), he or she may be reluctant to adopt that technology. Prior research has demonstrated that compatibility beliefs impact adoption decisions [36,47,48]. In the context of consumer health technologies, it is possible that a consumer has no issues with using a particular technology for non-health care purposes, but believes that the technology is not appropriate to use for health-related tasks. For example, impropriety beliefs may emerge if consumers are particularly concerned about whether the technology is sufficiently secure to protect sensitive health-related information.

Finally, it may be that a consumer believes that a technology is suitable for health-related tasks and that he or she has the ability to use the technology without undue effort, but simply does not enjoy using the technology. For example, many consumers may have the ability to use call centers and believe that call centers are appropriate for health-related tasks. However, these same consumers may not enjoy using call centers [49]. (In fact, our results, presented later, support this contention.) Although perceived enjoyment has not received as much attention as the other beliefs discussed here, there are studies that demonstrate a link between perceived enjoyment and adoption of a technology [50-52].

In response to the aforementioned issues, we pose the following questions:

Research question 3: What knowledge and motivation barriers exist for baby boomers using various forms of technology tools for health purposes?

Research question 4: How do knowledge and motivation barriers that exist for baby boomers using various forms of technology tools for health purposes differ from younger adults and older adults?

Table 1 shows the specific knowledge and motivation belief-based barriers included in this study, along with citations for supporting research.

**Table 1:** Adoption process barriers to consumers adopting health technology.

Barrier		Supporting research
**Knowledge-based barriers**		
	Awareness of the technology and its purpose	Rogers [36], Tak [35]
	Not knowing how to use the technology	Rogers [36], Hsieh [53], Zeman [54], Katz [55], Moorman [21], Jones [41]
**Motivation-based barriers**		
	Finding the technology too difficult to use	Rogers [36], Hsieh [53], Moorman [21], Phang [56], Emani [46], Kim [57], Jones [41]
	Needing more training on the technology’s use	Rogers [36], Hsieh [53]
	Technology is not sufficiently secure	Matthews [58], Phang [56], Zulman [59]
	Technology is not appropriate for health care use	Rogers [36], Karahanna [60], van Slyke [61], Emani [46]
	Do not enjoy using the technology	Hsieh [53], van Der Heijden [62], Thong [63], Brown [64]

## Methods

### Overview

The readiness/barrier instrument used in this study (see Multimedia Appendix 1) was part of a larger consumer health technology survey. The University Institutional Review Board of the first author approved the study as an exempt study. A paper-based and an online version of the survey were developed in parallel. The online version was custom-developed using Qualtrics software (Qualtrics Corp, Provo, UT, USA). The survey was developed in both formats for convenience and to allow participation by those who were and were not regular technology users. Participants were allowed to choose their preferred method of responding.

Demographic data were collected for each respondent at the end of the survey. The consumer health technology readiness/barrier instrument was located in the middle of the survey. The instrument asks 8 questions about the use of 11 types of technology for health purposes (phone, website, email, call center, video conference, texting, podcast, kiosk, smartphone, blog, and wiki). One question asked respondents’ perspectives concerning their readiness to use a specific type of technology and the remaining 7 questions addressed barriers to use of the same technology for health purposes.

To address research questions 1 and 2 (regarding respondents’ readiness to use technology for health purposes) respondents were asked to check a box by each type of technology if they agreed with the statement: “Would have no problem using this technology for health care.” This question stem is similar to that used in past research to ascertain that no barriers exist [41].

To address research questions 3 and 4, respondents were instructed to check a box by any issue (ie, barrier) they would face in using the specified type of technology to learn more about or manage their health (see  Multimedia Appendix 1 for specific instructions). The list of 7 barriers was informed by aggregating the knowledge-based and motivation-based technology barriers reported and assessed in prior studies (see Table 1 for the list of supporting research). The barrier statements were as follows: (1) don’t know what this technology is, (2) don’t know how to use this technology, (3) this technology would be too difficult to use, (4) would need more training on how to use this technology, (5) don’t feel the technology is secure, (6) doesn’t seem appropriate for health care purposes, and (7) don’t enjoy using this technology. Respondents were allowed to check multiple barriers. To show deliberate intent for those not selecting any barriers, respondents were asked to check a box indicating they would have no problem using a specific type of technology only if they checked none of the barriers.

Before the full data collection, a pilot study was conducted to assess the survey and data collection methods. A convenience sample of 14 individuals of varying ages completed 1 of the versions of the survey (7 on paper and 7 online) and then verbally answered a number of questions regarding the survey in general and specific survey items within each section of the survey. The pilot effort confirmed that the online version of the survey was functioning without error. Pilot participants of both the online and paper-based versions indicated that the parsimonious presentation (table format) of the barrier section of the consumer survey (1) was easy to understand, (2) provided a means for respondents to easily find and consider all barriers associated with a represented technology used for health care purposes as one holistic task, and (3) allowed respondents to quickly and efficiently answer the barrier section of the survey. Timed recordings indicated that the survey took between 20 and 35 minutes for respondents to complete and the method of data collection (paper vs online) did not seem to impact completion time. Feedback received from the pilot study led to minor modifications focused on enhancing readability and understandability.

Survey invitations and directions provided a layperson description of the consumer health technologies of interest to facilitate understanding of the survey context (see  Multimedia Appendix 1 for the types of consumer health technologies and the definitions provided in the survey). Respondents were able to select their preferred method of responding (paper vs online) as an attempt to increase the response rate. Offering a choice accommodated personal preference for survey method and also facilitated a means to participate for those that may not have ready access to a personal computer.

### Recruitment

The sample frame was composed of a sample of enrollees in a large pharmacy benefit management company. Participants who completed the survey were allowed to request that a small donation be made to a charitable organization on their behalf. The sampling frame afforded the opportunity to collect data from a national pool of respondents in the United States. The pharmacy benefit management company sent a personalized postal mail invitation to participate in the survey to a random sample of 3000 of their subscribers. The invitation included a paper-based copy of the survey as well as instructions for completing the online version as an option, if preferred. Only those who received the mail invitation were provided with the Web address for the online version of the survey. As a means to check for duplicate responses, paper-based surveys were stamped with unique identifiers and entry of this identifier was required as a log-in requirement to take the online version. One duplicate was identified and removed. Acceptable responses to the survey were provided by 506 individuals. However, age of the respondent was not available for 37 respondents. Therefore, data from 469 respondents were analyzed for this study resulting in a 15.63% response rate.

The surveys were completed from January 2010 through May 2011. Administration of the survey was timed to coincide with when the oldest boomers were reaching the retirement age of 65. The survey was initiated in a single state; thereafter, it was rolled out to other parts of the country. Reminders were sent by email at 3 months after the invitation was sent and then again at 7 months. It was not possible to send follow-up reminders specifically to nonrespondents, so only general reminders were sent that also directed respondents not to complete the survey a second time.

To understand respondents’ preferences for survey method, a binomial test was conducted to compare respondents’ selection of the paper versus the online version of the survey. Compared to an expected probability of 50%, respondents were significantly more likely to choose the paper version (59%, 277/469) over the online version (40.9%, 192/469; *P*<.001). Using the Kruskal-Wallis 1-way ANOVA on ranks test (H), age group differences for version of the survey completed were found to be statistically significant (H_2_=59.56, *P*<.001). All 3 post hoc pairwise comparisons using the Mann-Whitney *U* test were also found to be statistically significant (*P*<.05). Compared to baby boomers, the younger age group was significantly more likely to choose the online version (H_1_=–39.46, *P*=.03), whereas the older age group was significantly more likely to choose the paper version (H_1_=–76.50, *P*<.001). In the younger age group, 65.3% (47/72) completed the online version compared to 48.1% (124/258) of baby boomers and 15.8% (22/139) of the older age group. These results reinforce our decision to give participants a choice in how to respond. Had we used only paper-based or online surveys, it is likely that we would have introduced a systematic bias in our sample. Therefore, this study appropriately followed recommendations to provide choice regarding the survey response media for purposes of inclusiveness [65].

### Statistical Analysis

Frequencies and percentages were computed for the sample as a whole and by age group for relevant demographic, readiness, and barrier survey questions. Readiness and barrier survey questions used for this study were coded as yes/no responses. SPSS 20.0 (IBM Corp, Armonk, NY, USA) was used for all computations.

To address the first and second research questions regarding readiness to use the specified types of technology, the 11 types of technology were ordered from high to low based on the percentage of respondents that indicated they were ready to use each type. Kruskal-Wallis 1-way ANOVA on ranks test for independent samples was conducted for each of the 11 types of technology to test for age group differences. For these statistical tests, readiness responses were treated as a 2-point ordinal scales with unchecked responses coded as zero for “do not agree” and checked responses coded as 1 for “agree” in response to the statement “Would have no problem using this technology for health care.” For significant Kruskal-Wallis tests, pairwise post hoc comparisons were conducted using the Mann-Whitney *U* test. Bonferroni corrections were made to *P* values to protect against inflated type I error rate when carrying out multiple tests. In addition, eta squared (*η* ^*2*^) was used to estimate the effect size for significant omnibus and post hoc comparisons. Effect sizes <0.06 were considered small, ≥0.06 and <0.14 were medium, and ≥0.14 were considered large [66,67].

The third research question assessed respondents’ perceptions concerning 7 barriers to using the 11 types of technologies. A respondent could select more than 1 barrier. Although respondents were instructed to only check that they would have no problem using a specific type of technology if no barrier was checked, a few respondents (between 1 and 8 respondents for each type of technology) indicated that they had no problem using a technology and also checked a barrier to using that technology. For analysis, the number and percentage of respondents that checked at least 1 barrier were calculated for each type of technology. The types of technology were organized in a table format and ordered from low to high based on the percentage of total respondents that identified at least 1 barrier. The overall percentages for readiness and barriers for each type of technology were descriptively contrasted. Individual barriers identified for each type of technology by more than 14.9% (70/469) of the respondents were highlighted and described.

Analysis for the fourth research question regarding age group differences for barriers to technology use consisted of conducting chi-square tests on the raw nominal level data. Responses for the baby boomer age group were compared to responses of the younger and older age groups on the 7 barrier questions for each of the 11 types of technology. A total of 154 chi-square tests were computed for 2×2 contingency tables. That is, 77 chi-square tests compared baby boomers with the younger age group for each barrier and type of technology (7 barriers * 11 technologies=77 contingency tables) and 77 chi-square tests compared baby boomers to the older age group. For chi-square tests with expected cell counts less than 5 (ie, 59 contingency tables), the Fisher’s exact test was used instead of Pearson chi-square test. Only 2 contingency tables with expected cell counts less than 5 were statistically significant. Notably, low expected cell counts were more prevalent for comparisons between baby boomers and younger adults (eg, 40 contingency tables) because younger adults reported fewer barriers overall and were the age group with the smallest number or respondents.

## Results

### Demographics

Basic demographic data are presented in Table 2 for the sample as a whole (N=469) and by age group (younger age group: 72/469, 15.4%; baby boomer age group: 258/469, 55.0%; older age group: 139/469, 29.6%). For the baby boomer age group, responses were relatively gender balanced. This was not the case with the older and younger age groups. The younger age group had a significantly higher proportion of responses from females (males: 19/72, 26.4%; females: 53/72, 73.6%; *P*=.02) whereas the older age group had a significantly higher proportion of males (males: 61/139, 43.9%; females: 78/139, 56.1%; *P*<.001). Ethnicity distributions were similar for all age groups. Marital status and household income varied across the groups. These differences were not unexpected given the different life stages of the groups. For example, a significantly higher proportion of the younger group reported being single than was the case for the baby boomers (younger: 17/72, 26.6%; baby boomer: 17/258, 7.2%, *P*=.001) and the older group (younger: 17/72, 26.6%; older: 6/139, 4.5%, *P*=<.001).

Overall, the survey sample was healthy. Of the 436 respondents that answered the question “How would you rate your overall health?” 369 (84.6%) considered themselves to be in good to excellent health, 57 (13.1%) fair, and 10 (2.3%) poor. Of respondents that reported they were in poor health, 1 was in the older age group, 6 were in the baby boomer group, and 3 were in the younger age group.

Respondents were asked to indicate if they “would have no problems using [a specific type of] technology for health care...to learn more about or manage [their] health.” No particular health conditions were specified. As noted above, only 2% (10/436) of respondents considered themselves to be in poor health.

**Table 2:** Demographics of respondents (N=469).

Demographics		Age group, n (%)			Total
		18-45 (n=72)	46-64 (n=258)	>64 (n=139)	
Age (years), mean (SD)		35.3 (7.9)	56.9 (4.8)	73.2 (6.2)	58.4 (13.5)
**Gender**					
	Female	53 (73.6)	143 (55.4)	61 (43.9)	257 (54.8)
	Male	19 (26.4)	115 (44.6)	78 (56.1)	212 (45.2)
	Total responses	72	258	139	469
**Ethnicity**					
	Caucasian	57 (91.9)	198 (88.4)	115 (93.5)	370 (90.5)
	African-American	2 (3.2)	7 (3.1)	4 (3.3)	13 (3.2)
	Hispanic	1 (1.6)	8 (3.6)	2 (1.6)	11 (2.7)
	Asian	0 (0.0)	10 (4.5)	1 (0.8)	11 (2.7)
	Other	2 (3.2)	1 (0.4)	1 (0.8)	4 (1.0)
	Total^a^	62	224	123	409
**Marital status**					
	Single	17 (26.6)	17 (7.2)	6 (4.5)	40 (9.2)
	Married/partner	43 (67.2)	199 (84.3)	108 (81.2)	350 (80.8)
	Separated/divorced/widowed	4 (6.3)	20 (8.5)	19 (14.3)	43 (9.9)
	Total^a^	64	236	133	433
**Household income (US $)**					
	Under $30,000	1 (1.9)	13 (7.1)	24 (26.7)	38 (11.7)
	$30,000-$49,999	6 (11.5)	35 (19.0)	24 (26.7)	65 (19.9)
	$50,000-$99,999	19 (36.5)	80 (43.5)	29 (32.2)	128 (39.3)
	$100,000 or more	26 (50.0)	56 (30.4)	13 (14.4)	95 (29.1)
	Total^a^	52	184	90	326
**Health rating**					
	Excellent	8 (12.3)	21 (8.8)	8 (6.1)	37 (8.5)
	Very good	29 (44.6)	81 (33.9)	36 (27.3)	146 (33.5)
	Good	21 (32.3)	105 (43.9)	60 (45.5)	186 (42.6)
	Fair	4 (6.2)	26 (10.9)	27 (20.5)	57 (13.1)
	Poor	3 (4.6)	6 (2.5)	1 (0.8)	10 (2.3)
	Total^a^	65	239	132	436
**Survey version**					
	Paper	25 (37.7)	133 (51.6)	117 (81.5)	265 (56.5)
	Online	47 (65.3)	125 (48.4)	22 (15.8)	204 (43.5)
	Total	72	258	139	469

^a^Within-group differences in totals are due to missing responses.

### Boomer Readiness

As shown in Table 3, the percentages of all respondents that were ready to use each type of technology for health purposes varied widely—from a high of 82.7% (388/469) for use of the standard telephone to a low of 22.3% (105/469) for wikis. A scan of the distribution of percentages for all respondents revealed that the technologies fell into 3 clusters. Most of the respondents were comfortable with using the standard telephone, a health informational website, and email for health purposes—82.7% (388/469) to 73.8% (346/469) of respondents (the first cluster). Close to half of respondents (230/469, 49.0%) were ready to use automated call centers, medical video conferencing, and short message service (SMS) text messaging for health purposes—49.0% (230/469) to 42.0% 197/469) of respondents (the second cluster). The percentage of respondents that were ready to use technologies such as podcasts, personal digital assistant (PDA)/smartphone applications, blogs, and wikis for health purposes ranged from a high of 35.2% (165/469) to a low of 22.4% (105/469) (the third cluster). Looking at the distributions for the 3 age groups in Table 3, these technology clusters were more distinct for baby boomers than the younger or older age groups. Note that no cells had expected counts less than 5.

**Table 3:** Readiness to use consumer health technology by type of technology and age group.

Type of technology	Age group, n (%)			Total	Statistical tests		
	18-45 (n=72)	46-64 (n=258)	>64 (n=139)	(N=469)	H_2_	*P*	η^2^
Phone	57 (79.2)	222 (86.0)	109 (78.4)	388 (82.7)	4.4	.11	—
Website	65 (90.3)	218 (84.5) ^b^	83 (59.7)	366 (78.0)	39.7	<.001	0.09
Email	57 (79.2)	209 (81.0) ^b^	80 (57.6)	346 (73.8)	26.9	<.001	0.06
Call center	43 (59.7)	135 (52.3) ^b^	52 (37.4)	230 (49.0)	11.9	.003	0.03
Video conference	47 (65.3)	128 (49.6 ^b^	36 (25.9)	211 (45.0)	34.6	<.001	0.07
Texting	41 (56.9)	128 (49.6) ^b^	28 (20.1)	197 (42.0)	39.9	<.001	0.09
Podcast	41 (56.9)	95 (36.8 ^a,b^	29 (20.9)	165 (35.2)	27.7	<.001	0.06
Kiosk	37 (51.4)	91 (35.3) ^a^	33 (23.7)	161 (34.3)	16.3	<.001	0.04
Smartphone	41 (56.9)	89 (34.5) ^a,b^	16 (11.5)	146 (31.1)	48.6	<.001	0.10
Blog	38 (52.8)	74 (28.7) ^a^	26 (18.7)	138 (29.4)	26.6	<.001	0.06
Wiki	30 (41.7)	55 (21.3) ^a^	20 (14.4)	105 (22.4)	20.7	<.001	0.04

^a^Significantly different pairwise comparison between the baby boomer (46-64) and younger (18-45) age groups (*P*<.05).

^b^Significantly different pairwise comparison between the baby boomer (46-64) and older (> 64) age groups (*P*<.05).

### Readiness Comparisons Among Age Groups

In testing for age group differences, the Kruskal-Wallis H omnibus tests found the proportions for the 3 age groups were significantly different for all types of technology except the phone (see Table 3). Effect sizes were computed using eta squared to determine practical significance of these age group differences. Eta squared indicated a medium effect (0.06-0.10) for 7 types of technology (website, email, video conference, texting, podcast, kiosk, and blog) and a small effect (0.03-0.04) for 3 types (call center, kiosk, and wiki) (Table 3). The difference in percentages of respondents that indicated their readiness to use call centers for health purposes (η^2^=0.03) was only 8% between the younger and baby boomer groups and 15% between the baby boomer and older age groups. Similarly, the percent differences for kiosks (η^2^=0.04) was only 16% between the younger and baby boomer groups and 11% between baby boomer and older age groups. For wikis (η^2^=0.04), the percentage for the younger age group was double (42% vs 21%) the percentage for baby boomers whereas the difference between the baby boomer and older age groups was only 7%.

Post hoc pairwise comparisons conducted to determine which groups were statistically significantly different found differences between the younger and older groups for all 10 types of technology. Given that the focus of this study is on baby boomers, however, only the results from pairwise comparisons with baby boomers are annotated in Table 3 (significant pairwise comparisons are annotated with an “a” for younger and baby boomer comparisons and “b” for older and baby boomer comparisons) and discussed subsequently.

Based on the statistical and practical significance of post hoc comparisons, the 10 types of technologies fell into 2 clusters of 5 each. The first cluster included websites, email, call centers, video conferencing, and texting. There were no significant differences between baby boomers and the younger age group in their readiness to use the technologies for health purposes in the first cluster. However, in comparisons with the older age group, baby boomers were significantly more likely to be ready to use these technologies for health purposes. For these differences, the *P* values were small (*P*<.001) and the effect sizes were large (η^2^=0.14-0.18) for all technologies in this cluster except the call center which had a medium effect size (*P*=.01, η^2^=0.09). As noted earlier, the effect size was small for the omnibus test for call centers and the percent differences were 8% (younger to boomer) and 15% (boomer to older).

The second cluster (which included the remaining 5 technologies) was less distinct, but still demonstrated the role of age in readiness to adopt specific technologies for health purposes. In comparisons with the younger age group, baby boomers were significantly less likely (1.5 to 2 times less likely) to be ready to use the technologies in this cluster for health purposes (ie, podcasts, kiosks, smartphone apps, blogs, and wikis). These differences were significant (*P*<.005) with large effect sizes (η^2^ ≥0.14) for all technologies except the kiosk with a medium (*P*=.03, η^2^=0.12). As with the call center, the effect size reported earlier for the omnibus test for kiosks (see Table 3) was small (0.04) and the between-group percent differences were 16.1% for younger to boomer and 11.6% for boomer to older comparisons. For the kiosk, the percent difference was smaller between the boomer and older age group (11.6% vs 16.1% between the boomer and younger) and for the call center the percent difference was smaller between the boomers and the younger age group (7.4% vs 14.9% between boomer and older age groups).

In examining comparisons with the older age group for technologies in the second cluster, baby boomers were significantly more likely to be ready to use podcasts with a medium effect size (*P*=.005, η^2^=0.10) and smartphones with a large effect size (*P*<.001, η^2^=0.14). On the other hand, baby boomers were no more likely than the older age group to be ready to use kiosks, blogs, and wikis. Because baby boomers were less likely than the younger age group, but more likely than the older age group to be ready to use podcasts and smartphones, these 2 technologies may be promising targets for consumer health technologies applications.

It should be noted that although statistically significant, the effect sizes for the call center and kiosk indicated that the age group differences for these 2 types of technology were of limited practical significance.

### Boomer Barriers

Not surprisingly, the technologies fell into the same 3 clusters for the barrier measures as they did for the readiness measure (presented in Table 3) based on total percentages of respondents that checked at least 1 barrier. Of the 469 respondents, most indicated they were ready to use the phone (82.7%, 388/469) for health purposes (see Table 3) and few respondents identified barriers (16.6%, 78/469) to using the phone for health purposes (see Table 4). Similarly, websites (78.0%, 366/469) and email (73.8%, 346/469) had similar proportions of respondents that indicated they were ready to use these technologies for health purposes (see Table 3) and relatively few respondents selected barriers to using these technologies: 20.8% (98/469) for websites and 26.6% (125/469) for email (see Table 4).

**Table 4:** Respondents with barriers checked by type of technology and type of barrier.

Type of technology	Barriers to consumer health technology adoption, n (%)^a^							
	Total	Knowledge-based		Motivation-based				
	Checked >1 barrier	Don’t know what it is	Don’t know how to use	Too difficult to use	Need more training	Not secure	Not appropriate	Don’t enjoy using
Phone	78 (16.6)	5 (1.1)	9 (1.9)	4 (0.9)	6 (1.3)	15 (3.2)	16 (3.4)	31 (6.6)
Website	98 (20.9)	22 (4.7)	30 (6.4)	4 (0.9)	26 (5.5)	16 (3.4)	9 (1.9)	23 (4.9)
Email	125 (26.7)	9 (1.9)	23 (4.9)	5 (1.1)	12 (2.6)	44 (9.4)	32 (6.8)	29 (6.2)
Call center	238 (50.7)	15 (3.2)	23 (4.9)	6 (1.3)	9 (1.9)	40 (8.5)	75 (16.0)^a^	119 (25.4)^a^
Video conference	251 (53.5)	35 (7.5)	74 (15.8)^a^	16 (3.4)	70 (14.9)	29 (6.2)	30 (6.4)	54 (11.5)
Texting	269 (57.4)	16 (3.4)	57 (12.2)	12 (2.6)	28 (6.0)	61 (13.0)	87 (18.6)^a^	91 (19.4)^a^
Podcast	302 (64.4)	74 (15.8)^a^	89 (19.0)^a^	9 (1.9)	64 (13.6)	27 (5.8)	49 (10.4)	79 (16.8)^a^
Kiosk	307 (65.5)	31 (6.6)	45 (9.6)	7 (1.5)	29 (6.2)	83 (17.7)^a^	96 (20.5)^a^	96 (20.5)^a^
Smartphone	319 (68.0)	48 (10.2)	107 (22.8)^a^	19 (4.1)	63 (13.4)	49 (10.4)	45 (9.6)	91 (19.4)^a^
Blog	331 (70.6)	52 (11.1)	88 (18.8)^a^	10 (2.1)	54 (11.5)	81 (17.3)^a^	113 (24.1)^a^	92 (19.6)^a^
Wiki	357 (76.1)	93 (19.8)^a^	92 (19.6)^a^	8 (1.7)	43 (9.2)	91 (19.4)^a^	112 (23.9)^a^	74 (15.8)^a^

^a^Percentages of respondents (N=469) above 15% are marked to highlight the highest concentration of barriers.

However, among all respondents, the percentages that indicated barriers for the other technologies were double and triple the percentages that selected barriers for the phone, website, and email: 16.6% (78/469) to 26.7% (125/469) compared to 50.7% (238/469) to 76.1% (357/469) (see Table 4). As can be inferred from Table 3, 51.0% (239/469) to 58.0% (272/469) of respondents indicated that they were not ready to use call centers, video conferencing, or texting for health purposes (the second cluster). By the same token, more than half (50.7%, 238/469 to 57.3%, 269/469) of respondents identified at least 1 barrier to using these technologies. The 2 barriers checked by the highest percentage of respondents for call center and texting were “don’t enjoy using” (25.4%, 119/469 and 19.4%, 95.8/469, respectively) and “not appropriate” (16.0%, 75/469 and 18.6%, 87/469, respectively). For video conferencing, the 2 barriers checked by the highest percentage of respondents were “don’t know how to use” (16%, 74/469) and “need more training” (14.9%, 70/469). These percentages indicate that respondents are willing to use video conferencing if they were trained on its use. The objections to call centers and texting may be harder to overcome. Examining age group differences may provide more insights to guide adoption efforts for these technologies.

The third cluster of technologies (podcasts, kiosks, smartphones, blogs, and wikis) fared poorly overall. The percentage of respondents that cited at least 1 barrier for this cluster of technologies was lowest for podcasts (64.4%, 302/469) and highest for wikis (76.1%, 357/469). In examining the top barriers to use, the motivation-based barriers stood out, especially enjoyment, which was identified as a problem by more than 14.9% (>70/469) of respondents for every technology in this cluster (see Table 4). In addition to enjoyment, appropriateness and security were among the top motivation-based barriers checked for kiosks, blogs, and wikis. The most notable knowledge-based barrier was “don’t know how to use” with 19.0% (89/469) to 22.8% (107/469) of respondents checked this barrier for every technology in this cluster except kiosks. In addition, 15.8% (74/469) of respondents indicated that they did not know what a podcast was and what a wiki was.

### Barrier Comparisons Among Age Groups

**Figure 2:**
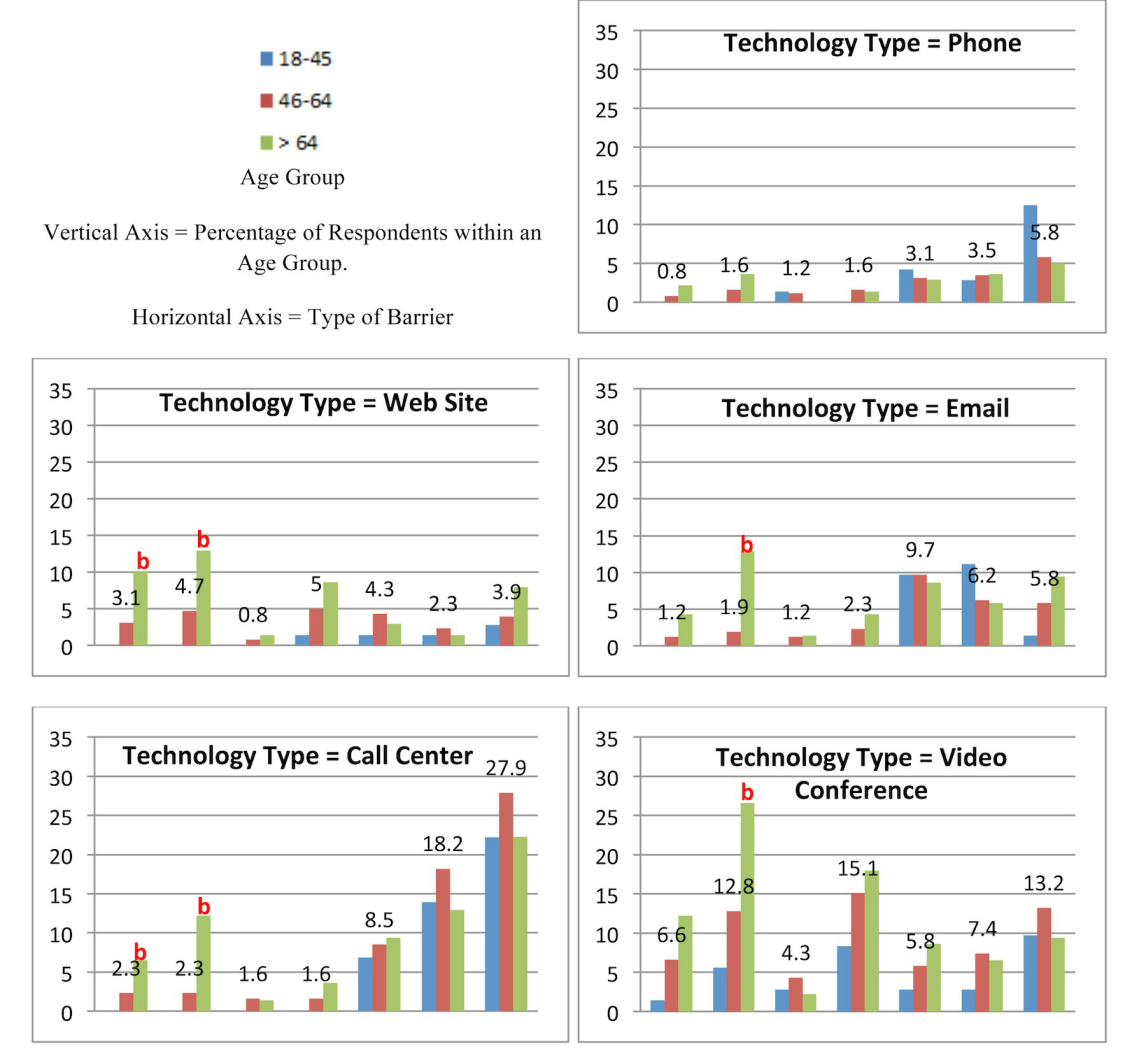
Bar charts of barriers to readiness to use consumer health technology by type of technology, type of barrier, and age group for phone, website, email, call center, and video conference.

**Figure 3:**
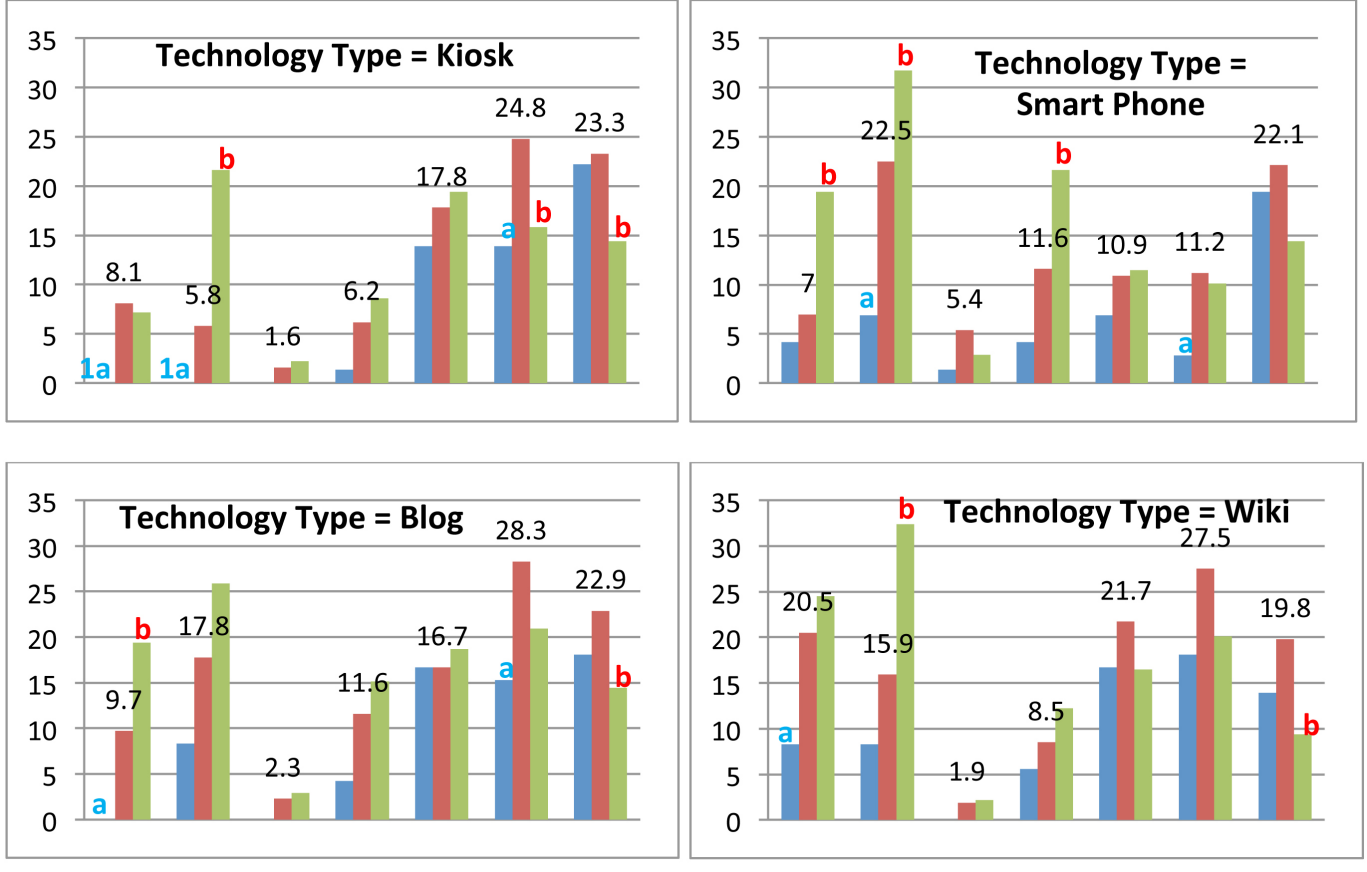
Bar charts of barriers to readiness to use consumer health technology by type of technology, type of barrier, and age group for texting, podcast, kiosk, and smartphone.

Chi-square tests comparing the baby boomer age group with the younger and older age groups yielded statistically significant results for 31 of the 154 pairwise comparisons (see Figures 2 and 3 for bar charts and Table 5 for actual counts and percentages). In all, 10 tests were statistically significant for the baby boomer and younger age group comparisons and 21 tests were significant for the baby boomer and older age group comparisons. Significant age group differences were found primarily among the knowledge-based barriers, 22 knowledge-based and 9 motivation-based. Consistent with the readiness findings reported earlier, the phone was the only type of technology for which there were no significant between-group differences for any of the barriers.

**Table 5:** Barriers to readiness to use consumer health technology by type of barrier, type of technology, and age group.

Type of tech by age group		Barriers to consumer health technology adoption, n (%)^a^						
		Knowledge-based		Motivation-based				
		Don’t know what it is	Don’t know how to use	Too difficult to use	Need more training	Not secure	Not appropriate	Don’t enjoy using
**Phone**								
	18-45	0 (0.0)^b^	0 (0.0)^b^	1 (1.4)^b^	0 (0.0)^b^	3 (4.2)^c^	2 (2.8)^c^	9 (12.5)
	46-64	2 (0.8)	4 (1.6)	3 (1.2)	4 (1.6)	8 (3.1)	9 (3.5)	15 (5.8)
	>64	3 (2.2)^b^	5 (3.6)^c^	0 (0.0)^b^	2 (1.4)^b^	4 (2.9)^c^	5 (3.6)^c^	7 (5.0)
**Website**								
	18-45	0 (0.0)^c^	0 (0.0)^c^	0 (0.0)^b^	1 (1.4)^c^	1 (1.4)^c^	1 (1.4)^c^	2 (2.8)^c^
	46-64	8 (3.1)	12 (4.7)	2 (0.8)	13 (5.0)	11 (4.3)	6 (2.3)	10 (3.9)
	>64	14 (10.1) ^d^	18 (12.9) ^d^	2 (1.4)^b^	12 (8.6)	4 (2.9)	2 (1.4)^c^	11 (7.9)
**Email**								
	18-45	0 (0.0)^b^	0 (0.0)^b^	0 (0.0)^b^	0 (0.0)^b^	7 (9.7)	8 (11.1)	1 (1.4)^c^
	46-64	3 (1.2)	5 (1.9)	3 (1.2)	6 (2.3)	25 (9.7)	16 (6.2)	15 (5.8)
	>64	6 (4.3)^c^	18 (12.9) ^d^	2 (1.4)^b^	6 (4.3)^c^	12 (8.6)	8 (5.8)	13 (9.4)
**Call center**								
	18-45	0 (0.0)^b^	0 (0.0)^b^	0 (0.0)^b^	0 (0.0)^b^	5 (6.9)	10 (13.9)	16 (22.2)
	46-64	6 (2.3)	6 (2.3)	4 (1.6)	4 (1.6)	22 (8.5)	47 (18.2)	72 (27.9)
	>64	9 (6.5) ^d^	17 (12.2) ^d^	2 (1.4)^b^	5 (3.6)^c^	13 (9.4)	18 (12.9)	31 (22.3)
**Video conference**								
	18-45	1 (1.4)^c^	4 (5.6)	2 (2.8)^c^	6 (8.3)	2 (2.8)^c^	2 (2.8)^c^	7 (9.7)
	46-64	17 (6.6)	33 (12.8)	11 (4.3)	39 (15.1)	15 (5.8)	19 (7.4)	34 (13.2)
	>64	17 (12.2)	37 (26.6) ^d^	3 (2.2)^c^	25 (18.0)	12 (8.6)	9 (6.5)	13 (9.4)
**Texting**								
	18-45	0 (0.0)^b^	3 (4.2)^c^	0 (0.0)^c^	1 (1.4)^c^	6 (8.3)	16 (22.2)	9 (12.5)
	46-64	6 (2.3)	13 (5.0)	7 (2.7)	13 (5.0)	38 (14.7)	51 (19.8)	56 (21.7)
	>64	10 (7.2) ^d^	41 (29.5) ^d^	5 (3.6)^c^	14 (10.1)	17 (12.2)	20 (14.4)	26 (18.7)
**Podcast**								
	18-45	3 (4.2) ^e^	5 (6.9)^e^	0 (0.0)^c^	6 (8.3)	3 (4.2)^c^	4 (5.6)	12 (16.7)
	46-64	38 (14.7)	44 (17.1)	7 (2.7)	38 (14.7)	16 (6.2)	28 (10.9)	52 (20.2)
	>64	33 (23.7) ^d^	40 (28.8) ^d^	2 (1.41)	20 (14.4)	8 (5.8)	17 (12.2)	15 (10.8) ^d^
**Kiosk**								
	18-45	0 (0.0) ^c,e^	0 (0.0) ^c,e^	0 (0.0)^b^	1 (1.4)^c^	10 (13.9)	10 (13.9) ^e^	16 (22.2)
	46-64	21 (8.1)	15 (5.8)	4 (1.6)	16 (6.2)	46 (17.8)	64 (24.8)	60 (23.3)
	>64	10 (7.2)	30 (21.6) ^d^	3 (2.2)^b^	12 (8.6)	27 (19.4)	22 (15.8)^d^	20 (14.4) ^d^
**Smartphone**								
	18-45	3 (4.2)^c^	5 (6.9) ^e^	1 (1.4)^c^	3 (4.2)	5 (6.9)	2 (2.8) ^e^	14 (19.4)
	46-64	18 (7.0)	58 (22.5)	14 (5.4)	30 (11.6)	28 (10.9)	29 (11.2)	57 (22.1)
	>64	27 (19.4) ^d^	44 (31.7) ^d^	4 (2.9)	30 (21.6) ^d^	16 (11.5)	14 (10.1)	20 (14.4)
**Blog**								
	18-45	0 (0.0) ^e^	6 (8.3)	0 (0.0)^b^	3 (4.2)	12 (16.7)	11 (15.3) ^e^	13 (18.1)
	46-64	25 (9.7)	46 (17.8)	6 (2.3)	30 (11.6)	43 (16.7)	73 (28.3)	59 (22.9)
	>64	27 (19.4) ^d^	36 (25.9)	4 (2.91)	21 (15.1)	26 (18.7)	29 (20.9)	20 (14.4) ^d^
**Wiki**								
	18-45	6 (8.3) ^e^	6 (8.3)	0 (0.0)^b^	4 (5.6)	12 (16.7)	13 (18.1)	10 (13.9)
	46-64	53 (20.5)	41 (15.9)	5 (1.9)	22 (8.5)	56 (21.7)	71 (27.5)	51 (19.8)
	>64	34 (24.5)	45 (32.4) ^d^	3 (2.2)^c^	17 (12.2)	23 (16.5)	28 (20.1)	13 (9.4) ^d^

^a^Percentage of respondents that indicated agreement with the barrier statement within an age group. Younger age group (18-45, n=72); baby boomers (46-64, n=258); older age group (>64, n=139).

^b^Contingency tables (n=24) containing 2 cells with expected cell counts <5.

^c^Contingency tables (n=35) containing 1 cell with expected cell counts <5.

^d^Significant pairwise comparisons (*P*<.05) between the boomer (46-64) and older ( >64) age groups (italicized).

^e^Significant pairwise comparisons (*P*<.05) between the boomer (46-64) and younger (18-45) age groups (italicized).

In baby boomer and younger age group comparisons, the younger age group was more favorable toward the technologies (ie, the younger group identified fewer barriers). Moreover, the comparisons between baby boomers and younger age group aligned closely with the readiness results reported earlier. As with the readiness measure, the technologies fell into 2 clusters. Consistent with the readiness findings, there were no differences between the baby boomer and younger age groups on the barrier measures for the first technology cluster (ie, website, email, call center, video conferencing, or texting). All 10 statistically significant findings between baby boomers and the younger age group were in the second technology cluster (podcasts, kiosks, smartphones, blogs, and wikis). Moreover, 7 of the 10 significant findings were for knowledge-based barriers. Baby boomers were significantly more likely to check “don’t know what it is” for all these technologies with the exception of the smartphone. They were significantly more likely than the younger group to check “don’t know how to use” for all technologies except for the blog and wiki. Three significant age group differences were found for 1 motivation-based barrier. Baby boomers were more likely than the younger age group to check “Doesn’t seem appropriate for health care purposes” for kiosks, smartphones, and blogs.

Comparisons between the baby boomer and older age groups indicate—although younger—baby boomers were not always more favorable toward the technologies. The trend toward favorability and younger age holds true for the first technology cluster (website, email, call center, video conferencing, or texting), but does not hold true for motivation-based barriers in the second technology cluster (podcasts, kiosks, smartphones, blogs, and wikis).

For the first cluster of technologies, baby boomers were generally more favorable to the technologies (ie, checked fewer barriers) than the older age group. Eight of the 21 statistically significant differences were among knowledge-based barriers in the first cluster. Compared to the older age group, baby boomers were significantly less likely to check knowledge-based barriers for website, call center, and texting. For these 3 technologies, baby boomers were 3 times less likely to check “don’t know what it is” and 3 to 6 times less likely to check “don’t know how to use.” For email and video conference, baby boomers and the older age group were equally likely to check “don’t know what it is” but the older ager group was significantly more likely to check “don’t know how to use.” Although there were no significant age group differences for motivation-based barriers in the first cluster, high percentages of all 3 age groups checked barriers related to appropriateness and enjoyment for call centers and texting. For videoconferencing, the motivation-based barriers were more related to training and enjoyment.

In the second cluster of technologies cluster (podcasts, kiosks, smartphones, blogs, and wikis), significant differences between baby boomers and the older age group were evenly split between knowledge-based (7 tests were significant) and motivation-based barriers (6 tests were significant). Of these 5 technologies, baby boomers’ responses were most favorable toward smartphones and podcasts. Baby boomers were significantly less likely than the older age group to check either of the knowledge-based barriers for these 2 technologies. For smartphones, only 7.0% (18/258) of baby boomers checked “don’t know what is” and 22.5% (58/258) checked “don’t know how to use” compared to 19.4% (27/139) and 31.6% (44/139) of the older age group. For podcasts, 14.7% (38/258) of baby boomers checked “don’t know what is” and 17.1% (44/258) checked “don’t know how to use” compared to 23.7% (33/139) and 28.8% (40/139) of the older age group. This finding is consistent with the earlier analysis on readiness measures and reinforces the argument that these 2 technologies may be promising targets for consumer health information technology applications for baby boomers.

There was less consistency for the other 3 technologies in the second cluster (kiosks, blogs, and wikis) around both knowledge-based barriers. Baby boomers were significantly less likely than the older age group to check “don’t know what it is” for blogs (9.7%, 25/258 vs 19.4%, 27/139N), but “don’t know how to use” for kiosks (5.8%, 15/258 vs 21.6%, 30/139) and wikis (15.9%, 41/258 vs 32.4%, 45/139). Importantly, a high percentage (over 20%) of both groups indicated that they don’t know what wikis were compared to 7.2% (10/139) to 8.1% (21/258) for kiosks.

Three motivation-based barriers were significant for technologies in the second cluster: “need more training on how to use,” “doesn’t seem appropriate for health care purposes,” and “don’t enjoy using.” Baby boomers were significantly less likely than the older age group to select the barriers related to training for the smartphone (11.6%, 30/258 vs 21.6%, 30/139). On the other hand, baby boomers were significantly more likely to check “not appropriate” as a barrier for kiosks (24.8%, 64/258 vs 15.8%, 22/139 and “don’t enjoy using” as a barrier for all technologies in this cluster except the smartphone (podcasts: 20.2%, 52/258 vs 10.8%, 15/139; kiosks: 23.3%, 60/258 vs 14.4%, 20/139; blogs: 22.9%, 59/258 vs 14.4%, 20/139; wikis: 19.8%, 51/258 vs 9.4%, 13/139).

In examining Figure 3 for smartphones, baby boomers were significantly less likely (7.0%, 18/258) than the older group (19.4%, 27/139) and equally as likely as the younger age group (4.2%, 3/72) to check “don’t know what it is.” Moreover, boomers (22.5%, 58/258) were significantly less likely than the older age group (31.7%, 44/139) and more likely than the younger age group (6.9%, 5/72) to check “don’t know how to use.” These findings indicate that baby boomers’ awareness of smartphones is on par with the younger age group, but they lag behind the younger group in knowing how to use smartphones. In fact, the 2 barriers checked by the highest percentage of baby boomers were “don’t know how to use” (22.5%, 58/258) and “don’t enjoy using” (22.1%, 57/258). Although none of the groups seemed to enjoy using smartphones for health purposes (younger: 19.4%, 14/72; boomers: 22.1%, 57/258; older 14.4%, 20/139), baby boomers and the younger age groups were significantly less likely than the older age group to indicate that they needed training. So although 22.5% (58/258) of baby boomers indicated they don’t know how to use smartphones for health purposes, enjoyment was a barrier for a higher percentage (22.1%, 57/258) than was training (11.6%, 30/258).

It is worthwhile to connect some of the readiness and barrier findings, particularly related to podcasts and smartphones. Recall from the readiness findings, podcasts and smartphones were 2 technologies baby boomers were more ready to use than the older age group and less ready to use than the younger age group. In examining the bar chart for podcasts in Figure 3, baby boomers were significantly less likely to check both of the knowledge-based barriers and significantly more likely to check “do not enjoy” using podcasts compared to the older age group. Podcasts were typically audio-only at the time of the survey. The ability to add images and video (ie, webcasting and streaming video) might increase enjoyment of podcast-like technologies.

## Discussion

### Principal Findings

Our results offer a number of insights that may be useful to those interested in designing and promoting the use of consumer health technologies. First, from our data it seems that baby boomers are ready to use numerous technologies for health-related purposes. Familiarity is a reasonable explanation for this readiness. Our data indicate that most baby boomers are ready to use the phone, websites, and email for health-related purposes. Independent data indicate that most baby boomers have experience with these technologies. A Pew Research report indicates that over 75% of baby boomers interact with websites and over 90% use email [30,40]. Familiarity with a technology is an important factor in determining subsequent adoption of that and related technologies. It is useful to think of consumer health technologies as clusters of technologies in which a core technology, such as a website, is adapted for a specific purpose such as retrieving health information. Familiarity with the core technology reduces the perceived risk of adopting the specific application of the technology [68]. Adoption of a new technology is by its very nature an uncertain venture; familiarity reduces this uncertainty, which increases readiness. Approximately 50% of baby boomers in our study indicated they were ready to use call centers, video conferencing, and texting for health care purposes and they were on par with the younger age group indicating only limited barriers to use. These findings indicate that the perceived risk of adopting these technologies for health care may be low for baby boomers.

When potential adopters are not familiar with the core technology, the costs of adoption are increased. This occurs primarily through 2 mechanisms, the first of which is the previously mentioned adoption risk. The second way concerns the costs of learning to use the new technology. If one is familiar with the core technology, the learning curve is largely limited to using the core technology for the new purpose. Consider the example of someone evaluating the use of email to communicate with a health care professional. If this individual is already familiar with using email in other contexts, he or she must only learn aspects of email use related to the health care context (eg, what information is appropriate to communicate via email).

Our thinking regarding the role of familiarity is confirmed by results related to consumer health technologies with low readiness. For example, only 37% of our baby boomer respondents reported being ready to use podcasts for health purposes. Pew Research reports that only 20% of baby boomers have interacted with podcasts [40]. Similar results are evident for smartphones. Less than 35% of our baby boomers reported being ready to use smartphones for health purposes. A Pew Research report from the same period as our data collection found that less than 30% of baby boomers used smartphones [69]. Although smartphones are often used for work purposes, smartphone adoption among older adults has been shown to lag behind that of younger consumers [70]. Findings related to blogs further confirm the role of familiarity. Approximately 29% of our baby boomers were ready to use blogs for health purposes, although Pew Research reported less than 30% of baby boomers use blogs [40]. (Comparative data are not available for video conferencing, kiosk, or wiki use.)

Comparisons across age groups are also explained by differences in familiarity. For example, approximately half of our baby boomer respondents indicated a readiness to use texting for health purposes compared with approximately 20% of our older group (age >64 years). According to a Pew Research report, over 70% of baby boomers use texting generally compared with 35% for the older age group [71]. Similar results were found with websites and email; 85% and 81%, respectively, of the baby boomers in our sample were ready to use these technologies for health purposes. The proportions were significantly lower for our >64 age group (60% and 58%, respectively). A Pew report indicated over 75% of baby boomers are online, compared with 58% of those aged 65-73 years and only 30% of those older than 74 years [40].

Our readiness results offer insights for change agents interested in promoting consumer health technology use. Change agents would be well advised to focus on technologies that are already familiar to sizable portions of the target age group. For example, sending older adults medication reminders through voice calls to mobile phones may be more effective than using smartphone notification systems, at least until smartphones become more widely adopted by the target group. Tablet computers (ie, iPads) offer an interesting example of this thinking. More seniors (aged ≥65 years) own tablets or e-book readers (27%) than own smartphones (18%), which is not the case with the general population [72]. Because of this, it may be more effective for change agents promoting consumer health technology use among seniors to focus on tablets rather than smartphones.

Our results indicate numerous significant differences in readiness across age groups. These results tell us that change agents should be cautious when extrapolating success in one age group to other age groups. Smartphones offer an example. Smartphone-based health applications may well be successful with younger consumers, but our results indicate that significantly fewer older consumers are ready to use smartphones for health applications. In fact, seniors are significantly less ready to adopt any technologies studied than their younger counterparts, with the exception of telephone voice calls.

This is not to say that we should completely eliminate from consideration any technologies with low readiness. As people age, as different age groups interact, and as the general adoption of core technologies improves, it is likely that readiness will improve. We advise keeping a close eye on the diffusion of the core technologies and timing the introduction and promotion of consumer health technologies applications to lag somewhat the diffusion of the core technology.

Change agents can also take steps to overcome a lack of familiarity. The role of trial use is helpful in countering unfamiliarity. Offering consumers easy, inexpensive ways to try an unfamiliar consumer health technology lowers adoption risk. Trials also allow consumers to experience the benefits of the consumer health technology, which will further increase the probability of adoption. Vicarious trials may also be beneficial. Vicarious trials are when another’s use of an innovation substitutes for one’s own trial use. These trials by close others may provide information that the potential adopter can use in evaluating the innovation [73]. It is important to choose the “other” carefully. Vicarious trials are more likely to be effective when the other party is an opinion leader [36] or is similar to the potential adopter or, in the case of health care, is a supporting caregiver.

Fundamentally, the decision to adopt or reject an innovation is typically based on an explicit or implicit cost-benefit analysis [36] in which the adopting unit (a consumer in this case) weighs the benefits of adopting an innovation against the perceived costs of doing so. Because of this, those interested in promoting adoption should understand how the potential adopter will view potential benefits and what the potential adopter will view as potential costs of adopting. These perceptions vary across individuals, and often across groups of individuals. In this paper, we focus on barriers to adoption readiness, which concerns the cost side of the adoption equation. In addition to monetary costs associated with adopting a consumer health technology, consumers also face real or perceived nonmonetary costs. These nonmonetary costs are the focus of our study.

Interestingly, the dominant barrier seems idiosyncratic to a particular technology. For example, in the case of kiosks, not being appropriate and not enjoying their use were the most frequently mentioned barriers (both 21%), but few participants reported not knowing how to use the kiosk as a barrier. In contrast, for smartphones, appropriateness was not an issue for a most respondents (10% reporting as a barrier), but not knowing how to use the technology was cited by 23% of the participants. Not surprisingly, the most frequently mentioned barriers for call centers were not being appropriate and not enjoying their use; few participants cited a lack of awareness, a lack of knowledge, or difficulty as being barriers to call center use. For consumer health technology designers and change agents, the message here is that it is important to not paint consumer health technologies with a broad brush with respect to barriers. The problematic barrier depends very much on the particular technology. Knowing which barriers apply to a particular consumer health technologies enables modifications in design or promotional activities that specifically address the dominant barriers. For example, making kiosks easier to use is not likely to substantially improve adoption. It would be more effective to address appropriateness. Further, it may be useful to consider patterns of barriers. Consider kiosks, blogs, and wikis. For all these technologies, security and appropriateness are frequently cited as barriers. It is possible that addressing the security barrier will also address the appropriateness barrier. More research is needed to verify this connection.

Turning attention to intergenerational differences in barriers (Figures 2 and 3), it is apparent that there is considerable variance across age groups in knowledge-based barriers. The main message from these findings is that age segmentation is important for those promoting use of consumer health technology. Seniors (aged >64 years) cite knowledge-based barriers more frequently than the other age groups for all technologies except voice phone calls. There are fewer differences in knowledge-based barriers between baby boomer and younger consumers. This is also true for motivation-based barriers. Awareness of intergenerational barriers is important for both consumer health technologies designers and change agents. Those who seek to promote consumer health technologies use to seniors must first address the knowledge-based barriers before turning attention to motivation-based barriers. This is especially important when the consumer health technology faces sizable awareness barriers. Awareness precedes persuasion in the innovation-decision process; consumers who are unaware of an innovation are unlikely to adopt that innovation.

Knowledge-based barriers seem to be less of an issue for baby boomers, particularly with respect to awareness. Not knowing how to use a technology is more of an issue for baby boomers than is a lack of awareness. Thus, change agents interested in promoting consumer health technologies use by baby boomers should focus more on helping consumers understand how to use the consumer health technologies. Appropriateness for health care use of certain technologies is also an issue for many of our baby boomer participants. Approximately 20% or more of baby boomers reported appropriateness as a barrier for texting, kiosks, blogs, and wikis.

The results of our research may serve as a starting point for future investigations. For example, the knowledge and motivation barriers we identified could be used in predictive models of actual adoption. Another interesting possibility is to compare groups within baby boomers, such as exploring gender differences, differences in household income, or differences across age groups within the boomer generation. Additionally, our findings could be used to inform consumer health technology design and promotional message selections, which could then be tested for their impact on adoption. Finally, future research should investigate how baby boomers use consumer health technologies, particularly in comparison to other groups, which may further facilitate the appropriate design of consumer health technologies targeted to specific groups. For example, a recent study found that baby boomers reported a significantly higher tolerance for having more Web components on a page than younger generations suggesting that younger generations would be more likely to miss key information if a Web page fails to present information using a limited number of clear focal points that are located on the first screen [51]. Such findings have not been assessed for health care websites.

### Limitations

As is the case with any study, there are a number of limitations to our research. First, we presented a limited number of barriers to technology use on the survey. Although our choices were based on the consumer health technologies and innovation adoption literature, these are evolving areas, so there may be important barriers that we did not investigate.

Second, there was no attempt to balance the number of respondents in each age group. Although demographic data on the sampling frame is proprietary and unavailable to us, we can extrapolate from the US Centers for Disease Control on prescription drug use and insurance coverage [74] and US Census data [75] to assess the representativeness of our data. To compute an expected distribution, we multiplied the population in each age group by the percentage in that population with health insurance. This gave us an expected number of individuals with health insurance. We then multiplied this number by the percentage of individuals in each group that take 1 or more prescription drugs. This gives us an expected number of people in each age group that both have insurance and take 1 or more prescription drugs. We then divided this number for each group by the sum of the groups to get the total proportion of individuals who have insurance and take prescription drugs. This yields a useful approximation of what proportion we should expect in each age group. Based on these calculations, it is likely that our sample overrepresents baby boomers (sample: 55% vs expected: 37%) and underrepresents the younger (sample: 15% vs expected: 22%) and older (sample: 30% vs expected: 41%) age groups. However, our underrepresentation of those aged 65 or older was still unexpected. Although this does not invalidate our results, it is an area of concern.

Third, the gender balance for the younger age group was skewed toward females, which was not the case with the other groups. To address the gender imbalance limitation, we computed chi-square statistics comparing “no problem” responses according to gender. The only significant difference (*P*<.10) was for wikis. Fifth, 39% of the contingency tables comparing consumer health technology barriers by age groups had small expected cell counts. We addressed this issue by using the Fisher’s exact test to test for significant differences, which should be kept in mind when interpreting our results.

Finally, caution should be taken in generalizing findings to uninsured populations. This sample was drawn from subscribers to a pharmacy benefit management company. Because the sample was drawn from enrollees in a pharmacy benefit management company, it is logical to assume all the respondents had some form of health insurance coverage. It is not known whether the respondent’s insurance company would pay for consumer health technologies used by the respondent. Furthermore, there are some implications that respondents may be able to bear some burden of consumer health technologies cost. Although income was not available for 31% of the sample, of those that reported income, 68% indicated they made over US $50,000 annually. In addition, 70% of the sample lived in the continental United States in a state east of the Mississippi River. This compares to 58% of the US population.

### Conclusions

Fulfilling the promise of consumer health technologies to impact health care cost and enable health care consumers requires adoption by health care consumers. Given their large numbers and growing health care needs, it is particularly important to understand what consumer health technologies baby boomers are ready to adopt. It is also important to understand what barriers block adoption for technologies with low adoption readiness. This paper addressed these issues.

Based on our analysis, most baby boomers are ready to adopt some types of consumer health technology (telephone voice calls, websites, and email). They were equally split on being ready to adopt call centers, video conferencing, and texting for health purposes. Baby boomers seem reluctant to adopt podcasting, kiosks, smartphone apps, blogs, and wikis. Specific adoption barriers vary according to the technology. For example, appropriateness and enjoyment seem to be the biggest barriers to adoption of call centers and texting, but knowing how to use and need for training are the biggest barriers for video conferencing. Further, baby boomers seem less ready to adopt some consumer health technologies than their younger counterparts, but are more ready to adopt than their elders. Differences between baby boomers and other consumers seem related to awareness, knowledge of how to use the technology, and the appropriateness and enjoyment of using technology for consumer health-related purposes.

Those interested in promoting use of consumer health technologies among baby boomers should consider these results when developing and choosing technologies, applications, and promotional tactics. Specifically, based on the results of this study, in combination with what is already known about innovation adoption, efforts to promote baby boomers’ use of consumer health technologies should focus on applications where the benefits most clearly outweigh the costs of adoption. That is, consumer health technologies that are (1) familiar to the baby boomers, (2) have clearly perceived benefits, and (3) require relatively little effort to use. Such an approach addresses both the benefit and cost sides of the adoption equation. Further, this approach is consistent with innovation adoption theory. Familiarity is associated with perceptions of compatibility. Easily communicated benefits increase perceptions of relative advantage and result in demonstrability. Low effort reduces perceptions of complexity. However, it should be noted that the relative strength of perceptions varies according to the adopter and the innovation.

## Multimedia Appendix 1

Health care barriers instrument.

## Abbreviations

SMS: short message service
