# Supplementary material for: Baby Boomers’ Adoption of Consumer Health Technologies: Survey on Readiness and Barriers
Source: J Med Internet Res. 2014 Sep 8;16(9):e200. doi: 10.2196/jmir.3049 (PMC4180340; doi:10.2196/jmir.3049)
Supplement: Supplementary file 1 [file jmir_v16i9e200_app1.pdf]

Multimedia Appendix 1. Health Care Barriers Instrument

There are many types of technology that are currently being used in health care. The table below contains some of these technologies and a description of the technology. The remaining columns allow you to indicate what, if any, issues you would face in using each technology. For each row, think about your potential use of each technology. It is ok if you have never actually used the technology. If you do not think you would have any issue in using the technology stated for health purposes, please check the first box “would have no problems using this technology for healthcare” and leave the other boxes in that row blank. If you do have issues with using the technology, mark ALL of the issues in the remaining columns that you think you would face in using each technology to learn more about or manage your health. Feel free to check more than one issue in each row.

Technology	Definition	Would have no problems using this technology for healthcare	Don’t know what this technology is	Don’t know how to use this technology	This technology would be too difficult to use	Would need training on how to use this technolo gy	Don’t feel the technology is secure	Doesn’t seem appropriate for health care purposes	Don’t enjoy using this technology
Blog	An online journal maintained by a person for health care purposes. A medical professional or a patient may write a blog.	1 0	2 0	3 0	4 0	5 0	6 0	7 0	8 0
Wikis	Web pages designed to enable anyone who accesses it to contribute or modify content related to health care.	1 0	2 0	3 0	4 0	5 0	6 0	7 0	8 0
Podcasts	A series of audio or audio-visual files related to health care that are distributed over the Internet and played through digital devices such as PCs and MP3 players.	1 0	2 0	3 0	4 0	5 0	6 0	7 0	8 0
Health Informational Website	Website accessed via the internet that contains information about health care issues.	1 0	2 0	3 0	4 0	5 0	6 0	7 0	8 0

Technology	Definition	Would have no problems using this technology for healthcare	Don't know what this technology is	Don't know how to use this technology	This technology would be too difficult to use	Would need training on how to use this technology	Don't feel the technology is secure	Doesn't seem appropriate for health care purposes	Don't enjoy using this technology
Medical Video Conferencing	Audio and visual telecommunications that patients use to interact with medical providers (for example, doctors and nurses) who are located someplace other than where the patient is located.	1 0	2 0	3 0	4 0	5 0	6 0	7 0	8 0
Applications on a PDA/Smart phone	Personal digital assistant or web-enabled mobile phone (for example, an Apple iPhone or Blackberry) that provides applications that can be used by patients to obtain health information, access personal health data, and record health-related data.	1 0	2 0	3 0	4 0	5 0	6 0	7 0	8 0
Voice Telephone Call	A standard person-to-person telephone conversation between patient and health care provider (for example, doctor or nurse or a pharmacist) to discuss health related topics.	1 0	2 0	3 0	4 0	5 0	6 0	7 0	8 0
Text Messaging	Sending "short" text messages from mobile telephones for health care purposes to or from health providers (for example, doctors or nurses).	1 0	2 0	3 0	4 0	5 0	6 0	7 0	8 0

Technology	Definition	Would have no problems using this technology for healthcare	Don’t know what this technology is	Don’t know how to use this technology	This technology would be too difficult to use	Would need training on how to use this technolo gy	Don’t feel the technology is secure	Doesn’t seem appropriate for health care purposes	Don’t enjoy using this technology
E-mail	An electronic mailing service used for communication between patient and medical provider (for example, doctor or nurse or pharmacist).	1 0	2 0	3 0	4 0	5 0	6 0	7 0	8 0
Automated Call Center	Computer generated calls from a centralized telephone system sponsored by an organization to provide health messages (such as reminders) or collect health data from a patient for medical record purposes.	1 0	2 0	3 0	4 0	5 0	6 0	7 0	8 0
Public Kiosk Applications	A touch-screen monitor placed in a public location that can be used by patients to access health-related information, such as information related to some form of health screening or to request a prescription refill.	1 0	2 0	3 0	4 0	5 0	6 0	7 0	8 0
